# Quantification of Human Photoreceptor–Retinal Pigment Epithelium Macular Topography with Adaptive Optics–Optical Coherence Tomography

**DOI:** 10.3390/diagnostics14141518

**Published:** 2024-07-15

**Authors:** Zhuolin Liu, Samira Aghayee, Somayyeh Soltanian-Zadeh, Katherine Kovalick, Anant Agrawal, Osamah Saeedi, Catherine Cukras, Emily Y. Chew, Sina Farsiu, Daniel X. Hammer

**Affiliations:** 1 Division of Biomedical Physics, Office of Science and Engineering Laboratories, Center for Devices and Radiological Health, US Food and Drug Administration, Silver Spring, MD 20993, USAsomayyeh.soltanian-zadeh@fda.hhs.gov (S.S.-Z.); anant.agrawal@fda.hhs.gov (A.A.); 2 Department of Ophthalmology, University of Maryland Baltimore School of Medicine, Baltimore, MD 21201, USA; osaeedi@som.umaryland.edu; 3 Division of Epidemiology and Clinical Applications, National Eye Institute, National Institutes of Health, Bethesda, MD 20892, USAechew@nei.nih.gov (E.Y.C.); 4 Department of Biomedical Engineering, Duke University, Durham, NC 27710, USA; sina.farsiu@duke.edu

**Keywords:** photoreceptor, retinal pigment epithelium, adaptive optics, optical coherence tomography, photoreceptor outer segment

## Abstract

Photoreceptors (PRs) and retinal pigment epithelial (RPE) cells form a functional unit called the PR-RPE complex. The PR-RPE complex plays a critical role in maintaining retinal homeostasis and function, and the quantification of its structure and topographical arrangement across the macula are important for understanding the etiology, mechanisms, and progression of many retinal diseases. However, the three-dimensional cellular morphology of the PR-RPE complex in living human eyes has not been completely described due to limitations in imaging techniques. We used the cellular resolution and depth-sectioning capabilities of a custom, high-speed Fourier domain mode-locked laser-based adaptive optics–optical coherence tomography (FDML-AO-OCT) platform to characterize human PR-RPE complex topography across the temporal macula from eleven healthy volunteers. With the aid of a deep learning algorithm, key metrics were extracted from the PR-RPE complex of averaged AO-OCT volumes including PR and RPE cell density, PR outer segment length (OSL), and PR/RPE ratio. We found a tight grouping among our cohort for PR density, with a mean (±SD) value of 53,329 (±8106) cells/mm^2^ at 1° decreasing to 8669 (±737) cells/mm^2^ at 12°. We observed a power function relationship between eccentricity and both PR density and PR/RPE ratio. We found similar variability in our RPE density measures, with a mean value of 7335 (±681) cells/mm^2^ at 1° decreasing to 5547 (±356) cells/mm^2^ at 12°, exhibiting a linear relationship with a negative slope of −123 cells/mm^2^ per degree. OSL monotonically decreased from 33.3 (±2.4) µm at 1° to 18.0 (±1.8) µm at 12°, following a second-order polynomial relationship. PR/RPE ratio decreased from 7.3 (±0.9) µm at 1° to 1.5 (±0.1) µm at 12°. The normative data from this investigation will help lay a foundation for future studies of retinal pathology.

## 1. Introduction

In the retina, the photoreceptors (PRs) and retinal pigment epithelium (RPE) act as a functional unit. RPE cells form a monolayer between Bruch’s membrane above the choriocapillaris (CC) and the PRs, but the apical surface of RPE cells have microvillae that wrap PRs and increase the surface area to promote nutrient and waste exchange via transepithelial transport between PRs and CC layers. In addition to this morphological coupling, the PR-RPE complex engages in several key aspects of visual function that exemplify its intertwined nature [1,2]. RPE is central to the visual cycle pathway for the regeneration of 11-*cis*-retinal from all-*tran*s-retinal produced during phototransduction, a capability lacking in PRs. Pigments (lutein, zeaxanthin, and lipofuscin) and melanin in the PR-RPE complex absorb light and protect against photo-oxidative damage via reactive oxygen species. The RPE promotes ion homeostasis in the subretinal space important for ion (K^+^, Na^+^, Cl^−^) exchange during the visual cycle. The PR and RPE also work closely together during photoreceptor outer segment (OS) renewal, where shed segments are digested, recycled in the RPE, and specific molecules returned to the PR for use in rebuilding OS disks, maintaining an overall constant OS length over the diurnal cycle. The RPE secretes a variety of growth factors important to maintain the structural integrity of the retina (e.g., pigment epithelium-derived factor, PEDF) and CC (e.g., vascular endothelial growth factor, VEGF).

The delicate symbiotic interplay in the PR-RPE complex is disrupted with disease. Inherited retinal degenerations (IRDs) involve mutations in genes for proteins essential to the visual cycle [3,4,5]. Atrophic (dry) age-related macular degeneration (AMD) involves the disruption of normal OS phagocytosis and enzymatic digestion in the RPE, leading to an accumulation of lipofuscin A2E, RPE cell loss, PR degeneration, and vision loss [6,7]. The dysfunction of growth factor secretion is involved in choroidal neovascularization in proliferative diseases like neovascular (wet) AMD [8]. Macular edema in diabetic retinopathy is, in part, a result of the disruption of the normal transport of water and glucose across the blood–retina barrier in the RPE and CC [9].

Only in the last few decades has a direct visualization of the PR-RPE complex in the live human eye become possible, first with optical coherence tomography (OCT), which provides high axial resolution and cross-sectional views amenable to retinal layer discrimination [10,11], and then with adaptive optics (AO)-enabled methodology, which corrects ocular aberrations for increased lateral resolution necessary to achieve cellular-level views. As the neurons where phototransduction starts, photoreceptors were the first cellular target for high-resolution in vivo human AO retinal imaging techniques [12,13] and continue to be studied extensively [14,15]. Only recently has the RPE mosaic been resolved in humans [16,17], a long-standing imaging challenge, owing primarily to the axial location of the RPE cell layer below the waveguiding and highly reflective photoreceptors (and incidentally, less from the size of the cells, which, at ~10–15 µm in diameter, are on scales accessible by non-AO methods). Various techniques have been introduced and demonstrated to increase the contrast of the RPE cell layer [18], usually in AO-enabled devices and with extensive image averaging. Visible (i.e., short wavelength autofluorescence, SWAF) and near-infrared autofluorescence (IRAF) adaptive optics–scanning laser ophthalmoscopy (AO-SLO) has been used to target endogenous melanin and lipofuscin localized only in RPE cells [19,20,21,22]. While melanin content is high in RPE cells, it also exists in the choroid, which can confound the RPE signal. Exogenous fluorophores like indocyanine green (ICG), long used for choroidal vascular visualization, have also been used to image RPE cells with AO (AO-ICG) when sufficient time has been allowed for the dye to be taken up by the cells [23]. ICG cellular uptake is variable and so AO-ICG images have a heterogeneous appearance that is localized to the RPE layer, providing cellular visualization. For reasons that are unclear, ICG dye appears hyper- or hypo-reflective in multiple adjacent cells, which makes the quantitation of individual cellular metrics somewhat difficult with AO-ICG. Non-confocal dark field AO-SLO methodology, which detects multi-scattered back-reflected light while suppressing single-backscattered ballistic photons, has also been demonstrated to resolve the RPE mosaic [24]. Dark-field AO-SLO offers no specific localized signal sensitivity with respect to RPE cells and typically provides the best contrast in the fovea and increasingly poorer images with increasing eccentricity, owing to scattering noise from other targets, particularly rod photoreceptors. Transscleral (vs. transpupillary) optical illumination combined with AO (AO-TIO) has shown excellent ability to rapidly resolve the RPE mosaic [25,26] using high oblique angle illumination to back-light RPE cells for improved contrast. AO-TIO is non-confocal and requires some high-pass filtering to remove out-of-focus light scattered from the choroid. AO-OCT takes advantage of the exceptional micron-scale depth sectioning capabilities of OCT to localize the signal to the RPE layer [27]. Because the contrast mechanism for AO-OCT RPE imaging is organelle motility, Liu et al. found optimum cellular contrast is achieved if volumes are acquired with a time interval close to the average decorrelation time of RPE organelles [28].

While PR density variation across the human macula is well documented and AO measurements have matched canonical histological studies [29], less has been reported on RPE morphological topography. Recent studies have begun to shed light on RPE topographical variations across the macula [21], including new models on how the PR-RPE complex relates to other structural characteristics like foveal shape [30] and the role it plays in diseases like AMD [31]. Despite this progress, in vivo human cellular-level retinal imaging is in a nascent stage and there are still significant discrepancies in structural parameter measurements (RPE density, PR/RPE ratio, and OSL) depending on the AO imaging methodology.

The aim of this study was to measure several key cellular topographical parameters across the macula from the PR-RPE complex using AO-OCT, which simultaneously produces high-contrast images of the PR and RPE layers extracted from high axial resolution volumes. The reproducibility of these AO-OCT measures was also assessed in a subset of the cohort. AO-OCT PR-RPE topographical measures may prove to be reliable biomarkers for use in treating retinal disease.

## 2. Materials and Methods

### 2.1. Approvals, Participants, and Eye Exam

Eleven participants, ranging in age from 27.7 to 42.9 years and free of ocular disease, were recruited for the study. Additional details about these participants are provided in Table 1. The study protocol was approved by the Institutional Review Board of the U.S. Food and Drug Administration (FDA). Written informed consent was obtained after the study procedures and potential risks were explained to each participant. Prior to the FDA AO imaging session, all participants underwent a comprehensive eye exam by the study ophthalmologists (OS and CC), including screening for fixational ability, ocular pathology, or contraindication to pupil dilation. For all participants who passed screening, the eye examination included the documentation of visual acuity (VA), fundus photography, biometry (IOLMaster 700; Carl Zeiss Meditec Inc., Dublin CA, USA), and OCT imaging (Spectralis, Heidelberg Engineering GmbH, Heidelberg, Germany).

Inclusion criteria were adult participants (>21 years old) who were able to understand and sign an informed consent and follow instructions during imaging. Exclusion criteria were participants who had any condition that prevented adequate quality images from being obtained (e.g., unstable fixation or media opacity), who had visual correction outside the range of +4 diopters to −8 diopters, who had a history of adverse reaction to mydriatic drops, who had a predisposition to (e.g., narrow iridocorneal angle) or history of acute angle closure glaucoma, or who worked under the direct supervision of the investigators.

### 2.2. AO-OCT Imaging

The custom-built FDA Fourier domain mode-locked (FDML)-based AO imaging system used in the study was previously described [32]. For this study, only data from the AO-OCT channel, which collects volumes at 13.0 Hz using the 3.4 MHz FDML swept source laser (λ_c_ = 1060 nm, ∆λ = 76 nm), were analyzed. The lateral and axial resolution of the system are estimated to be 2.9 µm and 8.4 µm, respectively.

**Figure 1:**
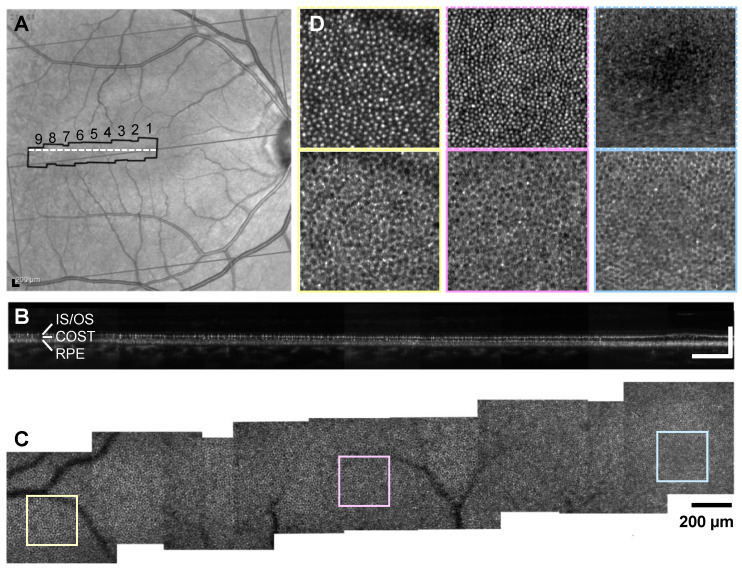
AO-OCT image acquisition example for a 33-year-old volunteer (8195). (**A**) Spectralis SLO image with nine AO-OCT imaging regions overlaid (black border). (**B**) Cross-sectional AO-OCT B-scan view of the PR-RPE complex along the temporal raphe at the region denoted by a white dashed line in (**A**). (**C**) Montage of RPE mosaics with AO-OCT. (**D**) Magnified 250 × 250 µm subregions of PR (top) and RPE (bottom) mosaic from three selected locations at fovea, 6T, and 12T. PR en face images were generated by average intensity projection across the inner segment/outer segment junction (IS/OS) and cone outer segment tip (COST) layers. The whole montage of PR and RPE of all study volunteers are provided in the Supplementary Materials. Scale bars = 200 µm.

One eye of each participant was imaged, and that eye was dilated and cycloplegia induced with Tropicamide 1%. After alignment in the system, the fixation target was set to direct the gaze of the participant to nine regions of interest (ROIs) from the fovea to 12° temporal (12T), where each imaging location was separated by 1.5° (Figure 1). At each location, three AO-OCT videos were collected with the field of view (FOV) set to 2° × 2° (a nominal overlap of 0.5° or 25%). Each video had 10 AO-OCT volumes separated by 6 s to allow for optimal contrast enhancement from RPE organelle speckle decorrelation [28]. The participants were instructed to blink naturally during the 60 s video duration.

Seven participants were re-imaged on two additional days for reproducibility assessment. Three videos (10 volumes each for 30 total volumes) were collected from two temporal locations (at the fovea and 7.5T) and averaged. These two locations were specifically chosen for quantification because RPE is known to have different melanin and lipofuscin concentrations that could affect RPE contrast at the locations sampled [33]. AO-OCT measurement reproducibility was assessed at these two locations over the three AO-imaging sessions.

### 2.3. Image Processing and Analysis

The 30 AO-OCT volumes collected at each ROI were processed, dewarped to correct for sinusoidal scanner motion, registered to correct for eye and head motion [34], averaged, and flattened to the PR/RPE complex. AO-OCT volumes collected during blinks or excessive eye motion were excluded. Post-processing was performed on the FDA High-Performance Computing (HPC) cluster which is a multi-user Linux-based computing environment comprising over 500 compute nodes, with 8–96 threads/node and 24 GB-2 TB of RAM/node. The RPE and PR layers were segmented separately (manually and semi-automatically, respectively) for further processing.

**Figure 2:**
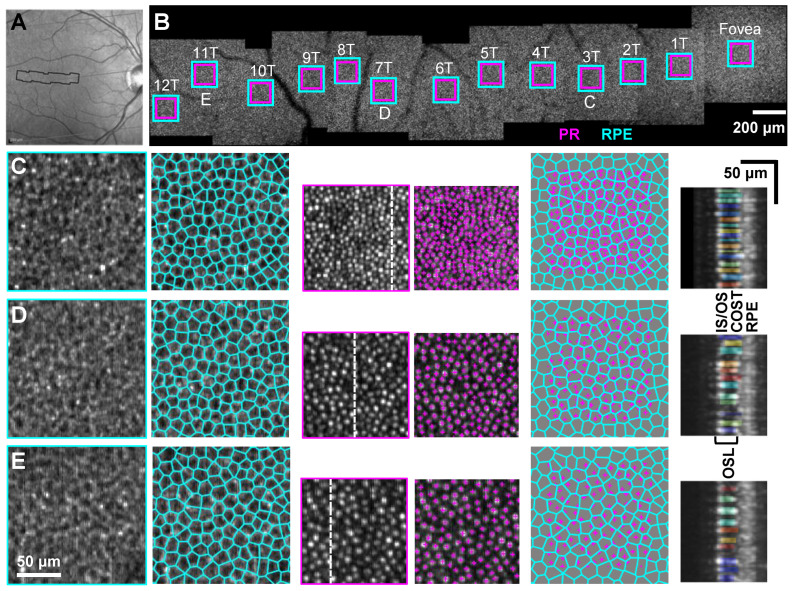
AO-OCT image processing and analysis approach for extracting outer retinal cell morphology. (**A**) Spectralis SLO image of a 32-year-old volunteer (1060) with AO-OCT imaging region overlay (black border). (**B**) AO-OCT montage of RPE mosaics showing 13 selected ROIs where PR-RPE complex quantification was performed. Magenta- and cyan-colored regions delineate areas used for cone photoreceptor and RPE quantification, respectively. For each ROI, the PR and RPE en face images are segmented and extracted from the average AO-OCT volume. Representative PR and RPE quantification results at (**C**) 3T, (**D**) 7T, and (**E**) 11T. For (**C**–**E**), images in rows from the left to right show RPE mosaic, Voronoi map with each RPE cell marked in cyan color, PR mosaic with cell centers marked in magenta color, superimposed RPE Voronoi map and PR locations, and segmented PR outer segments at the B-scans marked with the dashed white lines.

Outer retinal morphology was quantified at 13 selected ROIs from the fovea to 12T, with ~1° separation between adjacent selected regions (Figure 2). Cells from the segmented RPE layer (average of 2–3 axial pixels) were counted manually by two expert graders using custom software (Matlab, Mathworks, Natick, MA, USA). Cell markings were then processed using Voronoi analysis to derive RPE density and other metrics (cell-to-cell spacing, cell area). The values for the RPE metrics reported herein are mean values from the two graders. RPE cells were analyzed at all locations.

Cone PRs were counted from the AO-OCT volume using a previously reported automated machine learning (ML) algorithm [35]. After automated analysis, a single expert grader revised the counts with any necessary manual corrections to add cells missed and remove cells erroneously counted by the algorithm. The lateral locations for the corrected cells were then input back to the same ML software, which automatically measured the outer segment length (OSL) for each PR by identifying the cell reflections (peak signal) at the cone inner segment/outer segment junction (IS/OS) and cone outer segment tip (COST). The expert grader inspected the automatically segmented OSL results and if necessary, revised the identified reflections. Using the segmented COST layer, only cone PRs were counted in this study—no attempt was made to quantify rod PRs, which can be discriminated (both in the automated algorithm and the manual human correction) from the cone PRs, owing to the deeper rod outer segment tip (ROST) layer [27]. Final PR metrics extracted include PR density, PR OSL, and PR cell-to-cell spacing. PR-RPE ratios at each ROI are also reported. PR cone cells were analyzed at all locations except the fovea, where individual cells were not resolved with our AO-OCT system.

Both the RPE and PR density values excluded regions with vessel shadows, and both were scaled for each participant to correct for magnification differences due to the axial length using the Bennett eye model [36].

For the reproducibility measurements, the regions for analysis were selected and graded independently, where, due to subject fixational imprecision, no attempt was made to track or map individual PRs or RPE cells over the three AO-OCT imaging sessions for the seven volunteers examined. RPE counts were obtained as described above but from only one expert grader at the two locations examined (fovea and 7.5T). Measures of PR counts and OSL were obtained at the 7.5T location for the three AO-OCT imaging sessions using the ML algorithm, with manual correction by an expert human grader as described above.

### 2.4. Statistical Analysis

All statistical analysis was performed in Microsoft Excel with the Real Statistics Resource Pack and Analysis ToolPak add-ins, which include data analysis tools for statistical and engineering analysis. General statistical analyses included the calculation of the mean, standard deviation, and standard error. Paired *t*-tests and ANOVA (two-factor without replication) were used to analyze some measures. Regression analysis with Pearson (R_p_) or Spearman (R_s_) correlation was used to assess the relationship between eccentricity and PR density, RPE density, OSL, and PR/RPE using power, linear, and polynomial functions. Intraclass correlation coefficients (ICCs) were used to characterize the precision of measured inter-session parameters. ICCs were calculated using a two-way mixed effects model and a mean rating (k = 3) with 95% confidence intervals on absolute agreement. Measured ICC > 0.9 indicates excellent reliability, ICC = 0.75–0.9 indicates good reliability, ICC = 0.5–0.75 indicates moderate reliability, and ICC < 0.5 indicates poor reliability [37]. Lin’s concordance coefficient (LCC) was used to assess inter-rater variability between the graders for RPE counts [38]. The threshold *p*-value for statistical significance was set to 0.05.

## 3. Results

### 3.1. Primary PR-RPE Complex Metrics

**Figure 3:**
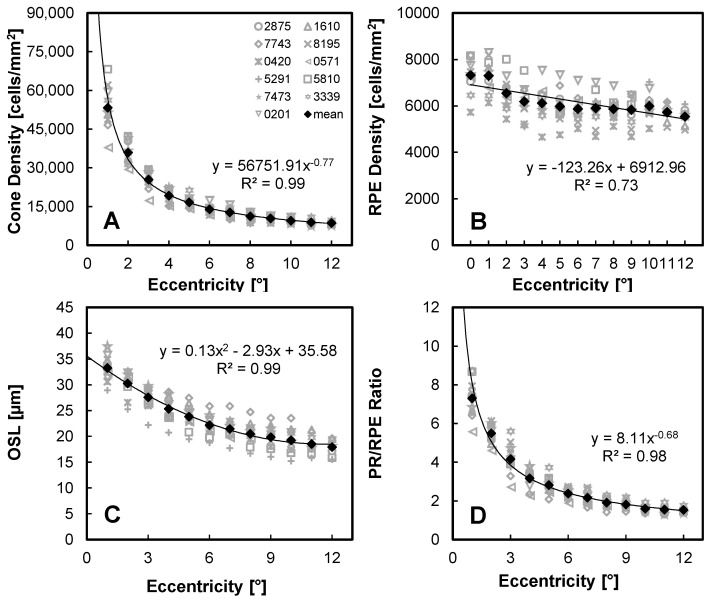
AO-OCT measurement of PR-RPE complex morphology with retinal eccentricity. (**A**) Cone PR density, (**B**) RPE density, (**C**) cone PR OSL, and (**D**) PR/RPE ratio. The black symbols are the mean values for our study cohort. The solid black lines represent the power/linear/polynomial fits to the mean data using Pearson’s correlation coefficient.

The morphological variation in the PR-RPE complex as a function of retinal eccentricity in the temporal retina for all study volunteers (gray symbols) and the overall cohort mean value (black symbols) is shown in Figure 3. Similar to other studies [30], the cone density followed a power function with eccentricity (Figure 3A, $R_{s}^{2}=0.93$; *p* < 0.05). The RPE density data were more variable, but a reasonable correlation was found using a linear relationship with eccentricity, where a negative slope of −123 cells/mm^2^ per degree from a peak at the fovea of 6913 cells/mm^2^ was observed (Figure 3B, $R_{s}^{2}=0.31$; *p* < 0.05). The slope of RPE density as a function of eccentricity was negative for all 11 healthy volunteers, with the slope ranging from −55 to −220 cells/mm^2^ per degree ($R_{s}^{2}$ ranging from 0.16 to 0.77 for the individual slope fits).

The relationship between OSL and eccentricity was best fit with a polynomial relationship (Figure 3C, $R_{s}^{2}=0.77$; *p* < 0.05). It should be noted that the OSL values reported herein are extracted from individually segmented cells and not merely from averaged retinal layer bands. As expected from the PR and RPE data, the PR/RPE ratio also followed a power law relationship with eccentricity (Figure 3D, $R_{s}^{2}=0.91$; *p* < 0.05).

### 3.2. Other PR-RPE Complex Measures

Power spectrum analysis has been used previously to rapidly assess the density of PR and RPE cells [18,27]. We compared Voronoi and power spectrum analyses (Figure S1) and found a reasonably good correlation between the measures ($R_{p}^{2}=0.79$). The slope of the fit was <1, indicating an underestimation of the power spectrum, as was previously observed [27], and could be attributed to an image windowing effect that causes the peak detection to skew toward lower frequencies (larger objects) and therefore lower densities. Secondary metrics including PR cell-to-cell spacing, RPE area, and RPE cell-to-cell spacing were also quantified (Figure S2). The RPE area and spacing were observed to increase gradually from the fovea to 6° and then plateau at high eccentricities to 12°.

There was very good agreement between the two graders for the RPE counts, with an LCC of 0.89 (95% confidence interval 0.85–0.92) for RPE density, 0.88 (95% CI: 0.83–0.91) for the RPE area, and 0.87 (95% CI: 0.82–0.90) for RPE spacing (Figure S3).

### 3.3. Reproducibility of PR-RPE Complex Measurements

**Figure 4:**
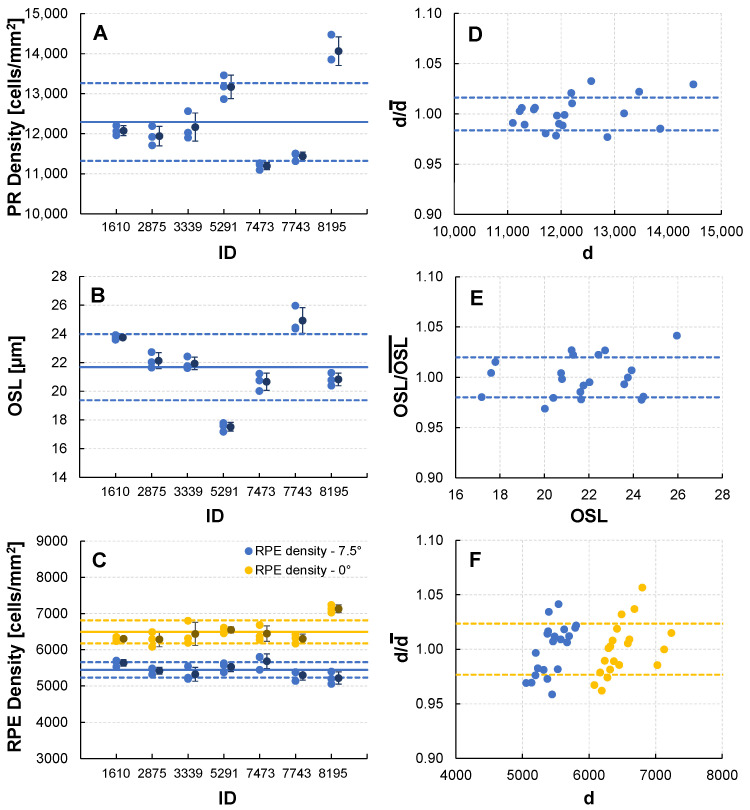
Reproducibility of AO-OCT PR-RPE complex measurements. (**A**–**C**) PR density, OSL, and RPE density measurements for three AO-OCT imaging sessions (light blue/orange symbols) and mean ± SD (dark-blue/brown symbol) for seven volunteers with overall cohort mean (solid line) and SD (dashed line). (**D**–**F**) Plot of normalized value ($x/\bar{x}$) vs. *x*, where $\bar{x}$ denotes mean value, illustrating the spread of repeat measurements for PR density (d), OSL, and RPE density (d) measurements. Dashed lines indicate overall cohort ±SD.

The mean separation between AO-imaging visits for the reproducibility cohort was 25 days (SD: 30, MIN: 2, MAX: 112). All measures showed low variance in repeat measurements, as illustrated in the individual participant measurements (Figure 4A–C), as well as the values normalized to the mean (Figure 4D–F). Excellent reproducibility was obtained for the two PR measures (PR density and OSL), with ICCs of 0.942 and 0.952, respectively. Owing to lower cell contrast, the ICC values for RPE density reproducibility were lower at 0.706 and 0.508 for the fovea and 7.5° regions, respectively. The overall standard deviation normalized to the mean was 1.6% for PR density, 2.0% for OSL, and 2.3% for RPE density (both locations).

## 4. Discussion

This study reports key PR-RPE complex measurements in the temporal macula of a healthy volunteer cohort using an AO-OCT imaging modality. Our observations represent the largest number of high-resolution, high-contrast AO-OCT images collected for this purpose, both in terms of sampled locations across the macula and also cohort size. We observed a tight grouping of cone PR density measurements following a power function with eccentricity, a more variable and linearly decreasing RPE density with eccentricity, a monotonically decreasing OSL with eccentricity, and a PR/RPE ratio following a power function with eccentricity.

**Figure 5:**
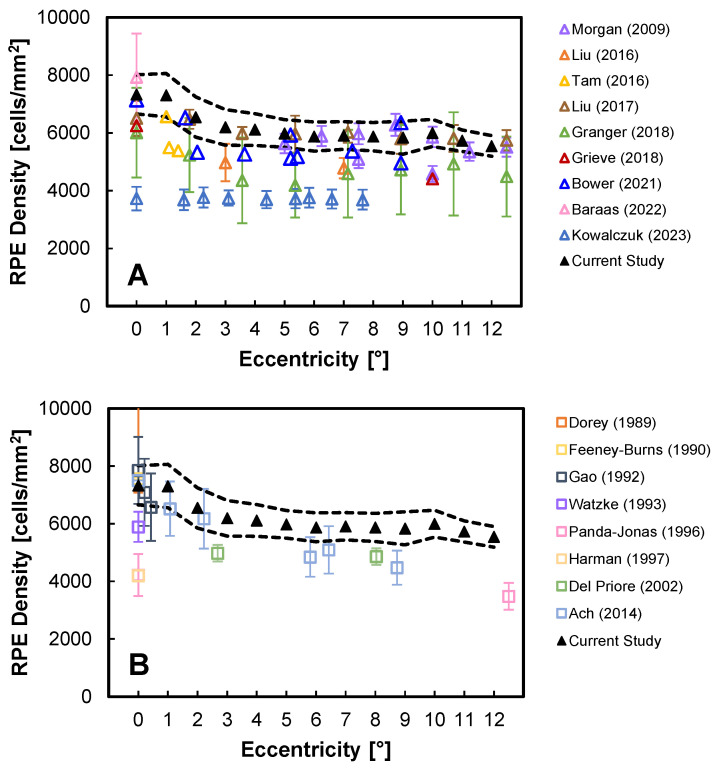
AO-OCT measurement of RPE density as compared to (**A**) in vivo studies [17,18,20,21,22,23,26,27,30] and (**B**) ex vivo studies [39,40,41,42,43,44,45,46]. The dashed lines denote ± SD from the mean.

Our RPE density results are compared with other human in vivo [17,18,20,21,22,23,26,27,30] and ex vivo [39,40,41,42,43,44,45,46] reports from the literature in Figure 5. In general, our data match previous AO-OCT single eccentricity values [18,27] and compare favorably with SWAF, IRAF, and dark-field AO-SLO [17,20,21,22,30] and AO-ICG results [23], albeit on the high side (Figure 5A). In particular, Granger et al. and Baraas et al. found the RPE density at the fovea to be 6008 and 7926 cells/mm^2^, respectively, matching reasonably closely to the range of the current study: 7335 (±681) cells/mm^2^. Granger et al. found a density of 4489 cells/mm^2^ at 12.5°, which was slightly lower than the 5755 (±852) cells/mm^2^ at 12° found in the current study. Kowalczuk et al. [26] reported much lower values (~4500 cells/mm^2^ at the fovea and ~3000 cells/mm^2^ at 15°T) and their slope with eccentricity was significantly shallower than our observations. This discrepancy is potentially because a low-pass filter was used by Kowalczuk et al. [26] to remove regions of smaller cells from their overall counts, which could have led to an underestimation of density, particularly at the fovea (lowering the slope).

Factors that may contribute to our slightly higher values than previous in vivo measurements include subject variability, age, and image modality. While subject variability is captured in our standard deviation range, which overlaps with most of the previously reported results (for variability ranges that were provided in previous reports), our cohort tended to be relatively young, which could have contributed to differences. Moreover, image modality differences, arising from different targeted sources of RPE cell contrast, could also contribute to different RPE density measurements. This methodology factor was recently explored by Bower et al. in a study imaging the same eyes with a multimodal adaptive optics imager [18].

In comparison to ex vivo values [39,40,41,42,43,44,45,46], our in vivo results match reasonably well for eccentricities <5°, except at the fovea, which has a wide range of reported values from ~4000–8000 cell/mm^2^ (Figure 5B). We report slightly higher RPE density values at eccentricities >5°, which may be attributed to age-related changes, spatial differences, or subject variability. Most previous ex vivo studies included an age range higher than ours and also measured lower RPE cell density for older eyes.

**Figure 6:**
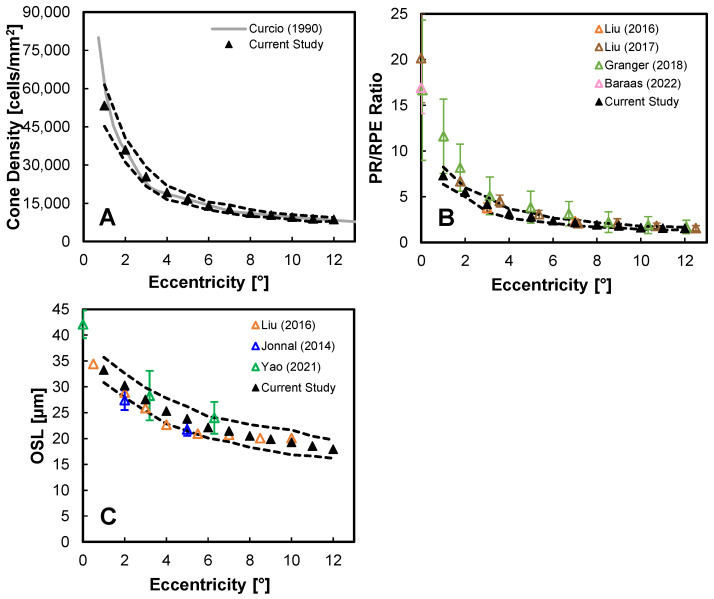
Comparison of AO-OCT measurements with published literature for (**A**) cone PR density [29], (**B**) PR/RPE ratio [21,22,27,30], and (**C**) PR OSL [27,47,48]. The solid line denotes mean values and the dashed lines represent ±SD for the entire study cohort.

The cone PR density, OSL, and PR/RPE density are compared with other human studies in Figure 6. The cone PR density compares favorably with the canonical results from Curcio et al. (Figure 6A) [29]. Owing to our generally higher RPE densities, we report PR/RPE ratios that are generally lower than previously reported (Figure 6B) [21,22,27,30].

PR OSL values as a function of eccentricity match previous AO-OCT studies [27,47], as well as clinical OCT data (Figure 6C) [48]. Our OSL values are measured from averaged volumes on a cone-to-cone basis using a previously published deep learning approach with manual correction [35]. For our data and those studies with similar results, we defined OSL as the distance between the peak intensity of the signal from the IS/OS and COST, which for AO-OCT images are paired beaded reflectance signals (see Figure 2) and for clinical OCT, owing to lower lateral resolution and averaging, are the second and third bands in the outer retina. Our results generally also compare well with histology, which found the OSL to be 35 µm in the central fovea (foveola), 28 µm in the parafovea (1–1.5 mm or ~5° eccentricity), and 20–23 µm in the mid-periphery (4 mm or 13.7° eccentricity) [49,50]. The distribution of PR OSL values for our cohort is shown for all locations in Figure S4, where most participants showed a consistent unimodal distribution at all locations, while a few (e.g., 0420) exhibited a bimodal distribution at some locations.

Our definition of the boundaries of the outer segments may not be consistent with some previous published studies, however, and controversy over the origin of the OCT signals in the outer retina remains [51,52,53]. Evidence to date, including animal studies, support the idea that the second and third bands in clinical OCT arise from the mitochondria in the ellipsoid region of the inner segments (ISe) and the interdigitation zone (IZ) between the photoreceptor outer segments and RPE [48,54]. However, the PR signals in AO-OCT are thinner and beaded, and the second signal appears closer to the third PR-RPE complex signal, indicating it may arise from a slightly different anatomical structure and axial location [47,48]. Other high-resolution OCT studies have used signal edges (the offset edge of the ellipsoid zone and onset edge of the phagosome zone) rather than peaks and reported lower values for the OSL [55]. Other ultrahigh-resolution OCT studies have reported higher values for the OSL [56]. If full consensus is achieved for the anatomical origins of OCT signals and the discrepancies between clinical OCT and AO-OCT are resolved, our OSL results can be updated using more precise axial locations from the averaged volumes.

AO-OCT achieves very good reproducibility for PR-RPE measurements. As expected from the relative cellular contrast, the PR measures had excellent reliability, while the RPE density reliability was lower, achieving moderate reliability at the fovea and 7.5°. The lower reliability for RPE reproducibility, particularly at 7.5°, could be attributed to lower image contrast from intrasession eye rotation, the effect of the molecular origin of the RPE signal (melanin vs. lipofuscin) at each location, as well as actual density variations across the macula for participants, since the regions were not registered across visits. Nevertheless, our results demonstrate that AO-OCT-based cellular metrics are excellent candidates for further development as retinal disease biomarkers.

Comparing imaging methodology [17,18,19,20,21,22,23,24,25,26,27,28] for PR-RPE complex topographical quantitation, AO-OCT shares advantages with other multimodal approaches in its ability to simultaneously image both the PR and RPE layers, but for AO-OCT this is accomplished by its extraction of layers and quantitative metrics from a single (averaged) volume. AO-OCT has significant advantages over other techniques for PR-RPE complex topographical quantification. Because of the micron-scale depth resolution afforded by the low coherence interferometry approach, AO-OCT volumes can be precisely segmented to visualize retinal layers and sub-layers, making AO-OCT more immune to crosstalk and interference from other structures of interest (e.g., PR signal imprinted on RPE images in dark-field AO-SLO). This also allows the simultaneous extraction of PR metrics like OSL, which is currently not possible without the µm scale depth sectioning capabilities of OCT. AO-OCT uses intrinsic organelle motility as a contrast mechanism, which compares favorably against techniques that require extrinsic dyes. Furthermore, AO-OCT RPE images are generated with significantly less averaging using organelle motion contrast than those generated with the relatively weak SWAF or IRAF fluorescence signals.

This study implemented several essential technical advances to allow the mapping of PR-RPE topography over the temporal macula for a relatively large cohort in contrast to the more moderate amount of data collected in previous AO-OCT RPE imaging studies [18,27,28]. The 3.4 MHz OCT acquisition speed provides high volume rates to significantly mitigate eye motion artifact and enables a 2° imaging FOV with sufficient lateral pixel density for cellular resolution. Our novel programmed saving scheme with variable volume time separation (6 s in the current study) improves the imaging efficiency by reducing the number of AO-OCT videos acquired from a few dozen to only three. Overall, we produced a total of 125 averaged AO-OCT volumes and quantified PR-PRE topography at 178 ROIs. This represents three times more information than that collected from all previous AO-OCT RPE studies, adding significant new, more comprehensive knowledge on PR-RPE cellular structure.

There are a few limitations to using AO-OCT for mapping PR-RPE topography. The use of organelle motility places a temporal (decorrelation) constraint on the acquisition time, particularly when significant averaging is required. A recent demonstration of AI to obviate the collection of multiple AO-OCT volumes for RPE imaging [57] may lessen the impact of this disadvantage. Also, as seen in our data, speckle noise and longer imaging wavelengths make the resolution of PRs in the foveola (central 0.25°) extremely difficult, and this is a task better suited for AO-SLO. Super-resolution techniques may eventually prove to be effective at overcoming this limitation [58]. While our device includes AO-OCT and AO-SLO channels [32], we did not collect small field videos from the foveola with AO-SLO to extract PR density values, and this is a limitation in our dataset.

## 5. Conclusions

The quantification of the topographical morphology of the PR-RPE complex in live human volunteers is a feat made possible only in the last few years with the help of AO methodology. The characterization of this important structure is important to better understand the anatomy of the normal retina, its variability across the population, for the construction of models of vision and visual pathways, as normative measures in comparison to eyes with retinal disease, in biomarker development, for other diagnostic purposes, and in the development of new therapies.

## Figures and Tables

**Table 1:** Summary of healthy participants.

ID	Age	Eye ^1^	Sex ^2^	Race ^3^	AL [mm]	Lateral Pixel Size [µm/pixel]
2875	32.8	OS	M	W	24.13	1.138
1610	32.0	OD	M	W	25.02	1.184
7743	42.9	OS	F	A	25.28	1.197
8195	33.3	OD	M	W	24.11	1.164
0420	27.7	OD	F	B	25.29	1.197
0571	42.2	OS	M	A	26.35	1.281
5291	36.6	OD	M	W	25.21	1.193
5810	36.5	OS	F	W	22.29	1.044
7473	36.6	OD	M	W	23.96	1.129
3339	36.6	OS	M	W	24.27	1.145
0201	32.9	OS	F	W	22.14	1.062
Mean ± SD	35.5 ± 4.4	6/5	4/7	1/2/8	24.37 ± 1.28	1.158 ± 0.066

^1^ Ratio of OS/OD, ^2^ ratio of F/M, ^3^ ratio of Black/Asian/White, AL: axial length.

## Data Availability

The original contributions presented in the study are included in the article/Supplementary Materials, and further inquiries can be directed to the corresponding authors.

## Supplementary Materials

The following supporting information can be downloaded at: https://www.mdpi.com/article/10.3390/diagnostics14141518/s1. Table S1: Summary of morphological characteristics of the outer retina measured by AO-OCT for all study volunteers; Figure S1: Correlation between RPE cell densities calculated from Voronoi and power spectrum analyses; Figure S2: Morphological quantification of PR cell-to-cell spacing, RPE cell area, and RPE cell-to-cell spacing; Figure S3: Intergrader agreement between two graders for RPE density, RPE cell area, and RPE cell-to-cell spacing; Figure S4: Histogram of PR OSL distribution for all participants at all locations; Figures S5–S15: AO-OCT PR and RPE mosaics in the temporal macula for all study volunteers.
