# Supplementary material for: Quantification of Human Photoreceptor–Retinal Pigment Epithelium Macular Topography with Adaptive Optics–Optical Coherence Tomography
Source: Diagnostics (Basel). 2024 Jul 15;14(14):1518. doi: 10.3390/diagnostics14141518 (PMC11276449; doi:10.3390/diagnostics14141518)
Supplement: Supplementary file 1 [file diagnostics-14-01518-s001.zip › diagnostics-3047297-supplementary.pdf]

Supplemental Material

for

Quantification of Human Photoreceptor – Retinal Pigment Epithelium Macular Topography with Adaptive Optics – Optical Coherence Tomography

Zhuolin Liu[1,*], Samira Aghayee[1], Somayyeh Soltanian-Zadeh[1], Katherine Kovalick[1], Anant Agrawal[1], Osamah Saeedi[2], Catherine Cukras[3], Emily Y. Chew[3], Sina Farsiu[4], and Daniel X. Hammer[1,*]

[1] Division of Biomedical Physics, Office of Science and Engineering Laboratories, Center for Devices and Radiological Health, US Food and Drug Administration, Silver Spring, MD 20993, USA; sam.aghayee@gmail.com (S.A.); somayyeh.soltanian-zadeh@fda.hhs.gov (S.S.-Z.); katherine.kovalick@fda.hhs.gov (K.K.); anant.agrawal@fda.hhs.gov (A.A.)

[2] Department of Ophthalmology, University of Maryland Baltimore School of Medicine, Baltimore, MD 21201, USA; osaeedi@som.umaryland.edu (O.S.)

[3] Division of Epidemiology and Clinical Applications, National Eye Institute, National Institutes of Health, Bethesda, MD 20892, USA; cukrasc@hotmail.com (C.C.); echew@nei.nih.gov (E.Y.C.)

[4] Department of Biomedical Engineering, Duke University, Durham, NC 27710, USA; sina.farsiu@duke.edu (S.F.)

* Correspondence: zhuolin.liu@fda.hhs.gov, Tel.: +1 301-796-7914; daniel.hammer@fda.hhs.gov, Tel.: +1 301-796-9320.

Table:

Table S1. Summary of morphological characteristics of the outer retina measured by AO-OCT for all study volunteers.

Figures:

Figure S1. Correlation between RPE cell densities calculated from Voronoi and power spectrum analyses on study cohort at all locations. The red dashed line denotes a slope of one, and the black solid line is the linear regression fit.

Figure S2. Morphological quantification for study cohort at all locations of **A.** RPE cell area, and **B.** RPE cell-to-cell spacing. Linear fit for RPE area and RPE spacing for 0-6° and 6-12° separately show monotonically increasing values to 6° and no further increase to 12°.

Figure S3. Intergrader agreement between two graders for study cohort at all locations for: **A.** RPE density (cells/mm^2), **B.** RPE cell area (μm^2), and **C.** RPE cell-to-cell spacing (μm). Perfect agreement is indicated by solid line.

Figure S4. Histogram of PR OSL distribution for all (healthy) participants at all locations. Magenta line is the mean value at each location.

Figures S5-S15. Heidelberg Spectralis SLO image (left) indicating temporal macula region images with AO-OCT (black border) and cone PR (top) and RPE (bottom) mosaics for all study volunteers.

Table S1. Summary of morphological characteristics of the outer retina measured by AO-OCT for all study volunteers.

**Cone Density [cells/mm²]**													
**Eccentricity [°]**	**2875**	**1610**	**7743**	**8195**	**0420**	**0571**	**5291**	**5810**	**7473**	**3339**	**0201**	**Mean [cells/mm²]**	**SD [cells/mm²]**
0													
1	52,841	50,721	46,588	62,171	48,825	37,968	52,008	68,266	51,765	56,299	59,166	53,328.9	8106.2
2	40,414	33,380	32,658	42,547	32,195	29,490	33,738	42,160	36,991	31,313	40,391	35,934.3	4716.8
3	25,154	24,764	21,963	29,736	23,277	17,379	23,711	29,309	28,608	28,897	27,243	25,458.3	3793.9
4	20,780	20,357	15,256	22,217	16,188	15,213	19,112	21,584	22,518	17,516	20,631	19,215.6	2739.4
5	17,487	15,882	14,331	16,204	15,103	14,229	15,711	17,873	17,224	21,331	17,751	16,647.8	2023.7
6	13,339	13,056	13,172	14,579	11,691	11,851	15,009	14,441	14,540	14,706	17,087	13,951.9	1542.9
7	11,967	13,870	10,053	13,299	10,713	10,860	12,878	13,145	12,770	14,545	15,437	12,685.2	1665.3
8	11,725	11,131	8621	11,501	8826	10,638	11,400	12,911	11,270	12,644	12,514	11,198.3	1405.6
9	10,256	9901	9431	10,717	8528		10,737	10,980	10,309	11,678	11,147	10,368.4	910.0
10	8701	9466	8447	10,033	8283		10,227	9607	8983	11,178	10,584	9550.9	959.5
11	7723	8629	8310	8212			10,161	8765	8099	10,924		8852.9	1109.0
12	8207	8098	8702				9666	8148	8121	9743		8669.3	737.1

**RPE Density [cells/mm²]**													
**Eccentricity [°]**	**2875**	**1610**	**7743**	**8195**	**0420**	**0571**	**5291**	**5810**	**7473**	**3339**	**0201**	**Mean [cells/mm²]**	**SD [cells/mm²]**
0	7044	7322	8182	7476	5728	7244	7921	8168	7409	6467	7724	7335.0	681.2
1	7083	7656	7260	8193	6143	6793	7166	7864	7503	6450	8273	7307.7	752.4
2	6798	6731	6410	6931	5439	6399	6058	8010	6099	5894	7266	6548.7	696.4
3	6156	6119	6672	5902	5219	6381	6306	7527	5997	5155	6672	6191.5	615.9
4	6150	5982	6511	6093	4664	6645	5863	6433	5860	5586	7510	6118.1	550.7
5	6295	6186	6881	5450	4761	5790	5909	5638	5866	5705	7314	5981.2	479.0
6	5902	5966	6335	5311	5029	6236	5987	5703	5647	5442	7076	5875.7	503.2
7	5859	6167	6022	4943	4685	6070	6198	6701	5836	5545	6990	5910.5	476.4
8	5713	6124	6070	5508	5122	6051	5623	6139	5890	5495	6838	5870.2	488.1
9	5617	5960	6507	4989	4679	6419	6383	6054	5855	5317	6436	5837.7	572.2
10	5838	5758	6256	5658	5027		7039	6056	5840	5780	6733	5998.6	466.5
11	5875	5290	6173	5092			6164	5733	5863	5670		5732.4	369.7
12	5819	5133	5598	4970			6081	5628	5645	5499		5546.8	355.7

**OSL [μm]**													
**Eccentricity [°]**	**2875**	**1610**	**7743**	**8195**	**0420**	**0571**	**5291**	**5810**	**7473**	**3339**	**0201**	**Mean [μm]**	**SD [μm]**
0													
1	33.2	34.8	32.8	34.9	30.6	32.3	28.9	33.7	37.6	31.6	35.7	33.3	2.4
2	30.7	31.5	30.9	30.1	26.6	31.4	25.3	29.6	32.1	32.7	32.2	30.3	2.4
3	29.2	29.4	29.2	27.4	25.9	26.4	22.2	26.4	30.0	28.5	28.8	27.6	2.3
4	27.1	27.9	28.6	24.7	23.6	23.7	20.7	23.6	28.3	24.3	26.0	25.3	2.5
5	25.3	26.4	27.5	22.7	23.1	22.9	19.5	20.8	25.0	24.6	24.5	23.8	2.4
6	22.3	24.2	25.9	21.4	21.2	21.1	18.8	19.8	24.3	22.0	23.0	22.2	2.1
7	20.3	22.8	25.9	20.5	22.1	19.8	17.7	20.4	22.2	23.1	21.3	21.5	2.1
8	21.3	22.5	24.7	19.5	19.4	19.7	16.7	17.9	21.2	21.2	21.3	20.5	2.2
9	20.2	22.2	23.6	19.1	18.0		16.1	17.6	21.7	20.1	20.2	19.9	2.3
10	19.2	21.4	23.5	18.6	17.4		15.2	16.8	19.1	20.7	20.6	19.3	2.4
11	17.6	21.3	20.8	18.1			15.8	16.7	18.0	19.8		18.5	1.9
12	17.2	19.5	19.6				15.6	15.8	18.4	19.6		18.0	1.8

**PR/RPE ratio**													
**Eccentricity [°]**	**2875**	**1610**	**7743**	**8195**	**0420**	**0571**	**5291**	**5810**	**7473**	**3339**	**0201**	**Mean**	**SD**
0													
1	7.5	6.6	6.4	7.6	7.9	5.6	7.3	8.7	6.9	8.7	7.2	7.3	0.9
2	5.9	5.0	5.1	6.1	5.9	4.6	5.6	5.3	6.1	5.3	5.6	5.5	0.5
3	4.1	4.0	3.3	5.0	4.5	2.7	3.8	3.9	4.8	5.6	4.1	4.2	0.8
4	3.4	3.4	2.3	3.6	3.5	2.3	3.3	3.4	3.8	3.1	2.7	3.2	0.5
5	2.8	2.6	2.1	3.0	3.2	2.5	2.7	3.2	2.9	3.7	2.4	2.8	0.5
6	2.3	2.2	2.1	2.7	2.3	1.9	2.5	2.5	2.6	2.7	2.4	2.4	0.3
7	2.0	2.2	1.7	2.7	2.3	1.8	2.1	2.0	2.2	2.6	2.2	2.2	0.3
8	2.1	1.8	1.4	2.1	1.7	1.8	2.0	2.1	1.9	2.3	1.8	1.9	0.2
9	1.8	1.7	1.4	2.1	1.8		1.7	1.8	1.8	2.2	1.7	1.8	0.2
10	1.5	1.6	1.4	1.8	1.6		1.5	1.6	1.5	1.9	1.6	1.6	0.2
11	1.3	1.6	1.3	1.6			1.6	1.5	1.4	1.9		1.5	0.2
12	1.4	1.6	1.6				1.6	1.4	1.4	1.8		1.5	0.1

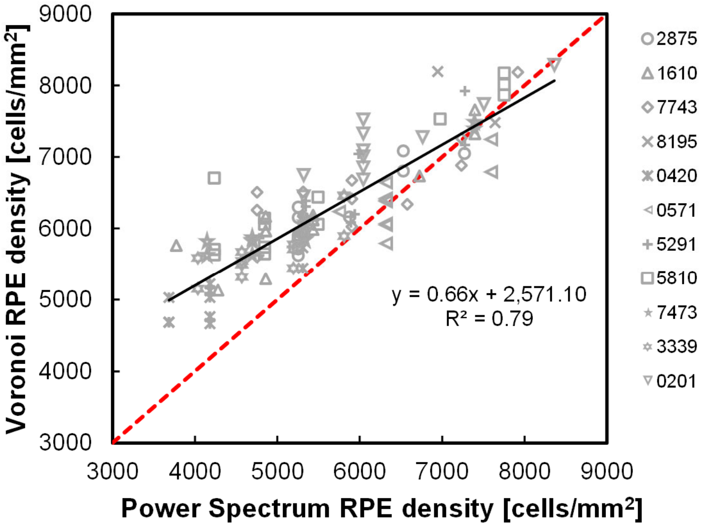

Figure S1. Correlation between RPE cell densities calculated from Voronoi and power spectrum analyses on study cohort at all locations. The red dashed line denotes a slope of one, and the black solid line is the linear regression fit.

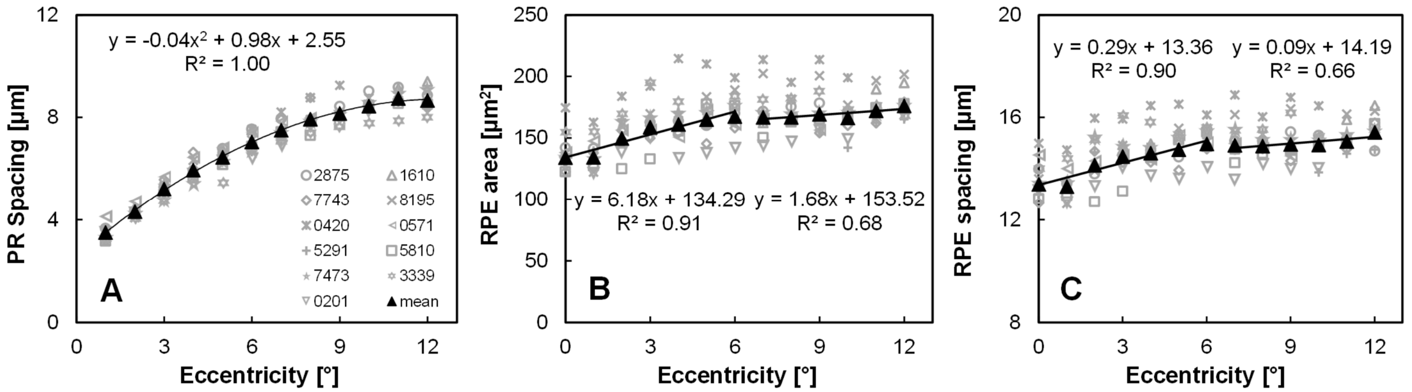

Figure S2. Morphological quantification for study cohort at all locations of **A.** PR cell-to-cell spacing, **B.** RPE cell area, and **C.** RPE cell-to-cell spacing. Linear fit for RPE area and RPE spacing for 0-6° and 6-12° separately show monotonically increasing values to 6° and only a slight increase thereafter from 6° to 12°.

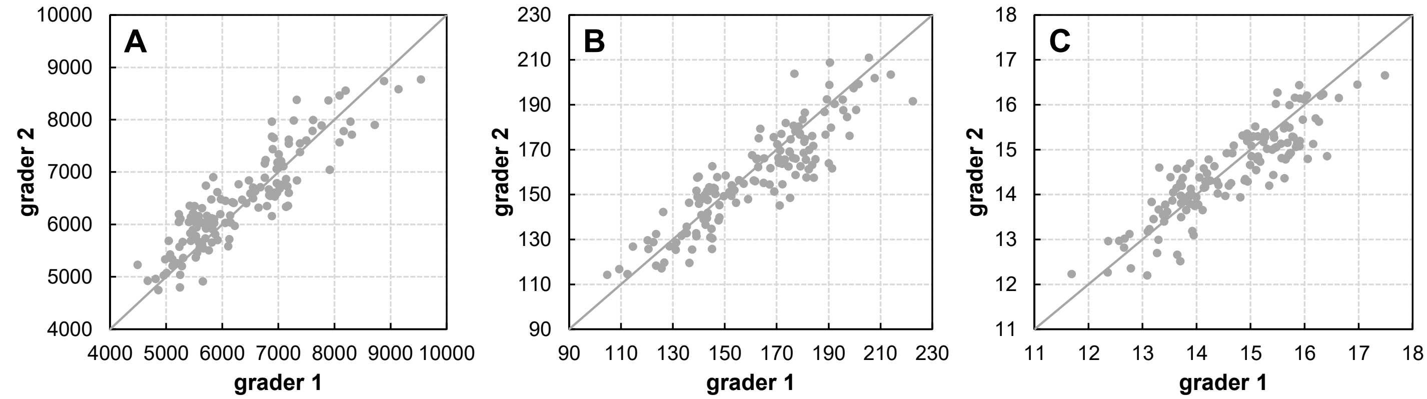

Figure S3. Intergrader agreement between two graders for study cohort at all locations for: **A.** RPE density (cells/mm²), **B.** RPE cell area (µm²), and **C.** RPE cell-to-cell spacing (µm). Perfect agreement is indicated by solid line.

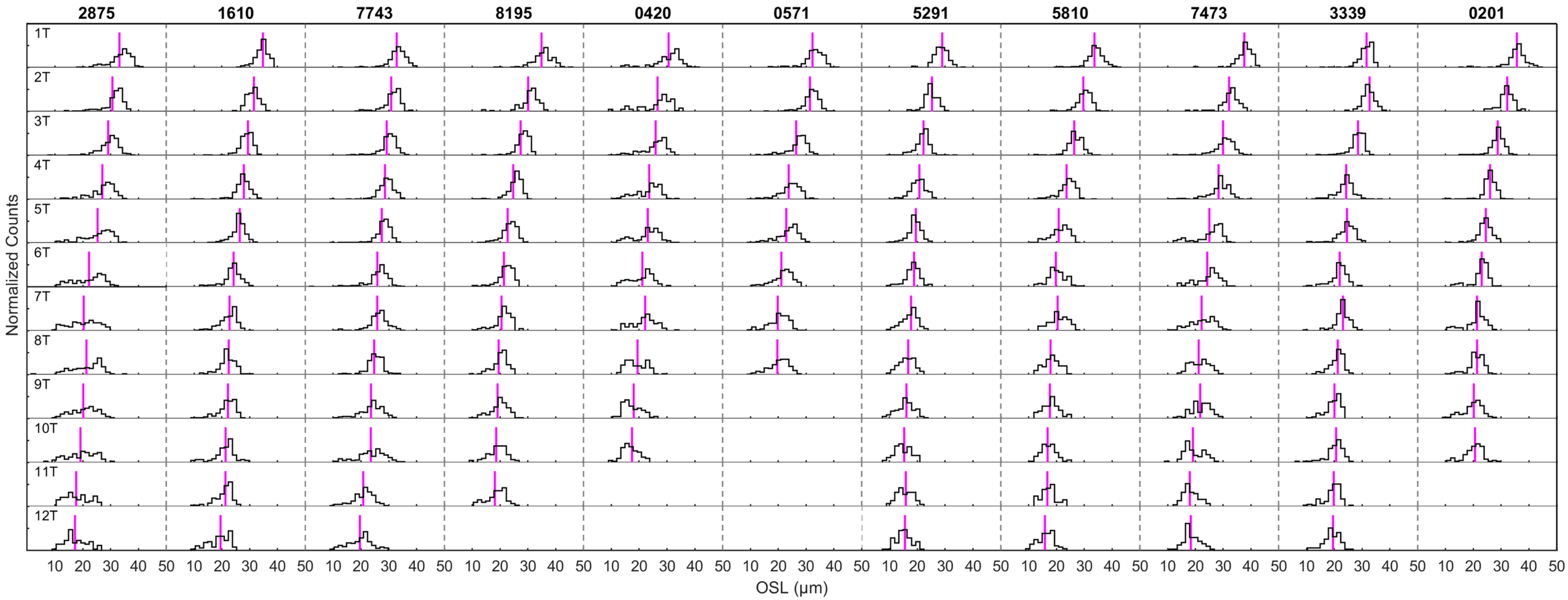

Figure S4. Histogram of PR OSL distribution for all (healthy) participants at all locations. Magenta line is the mean value at each location.

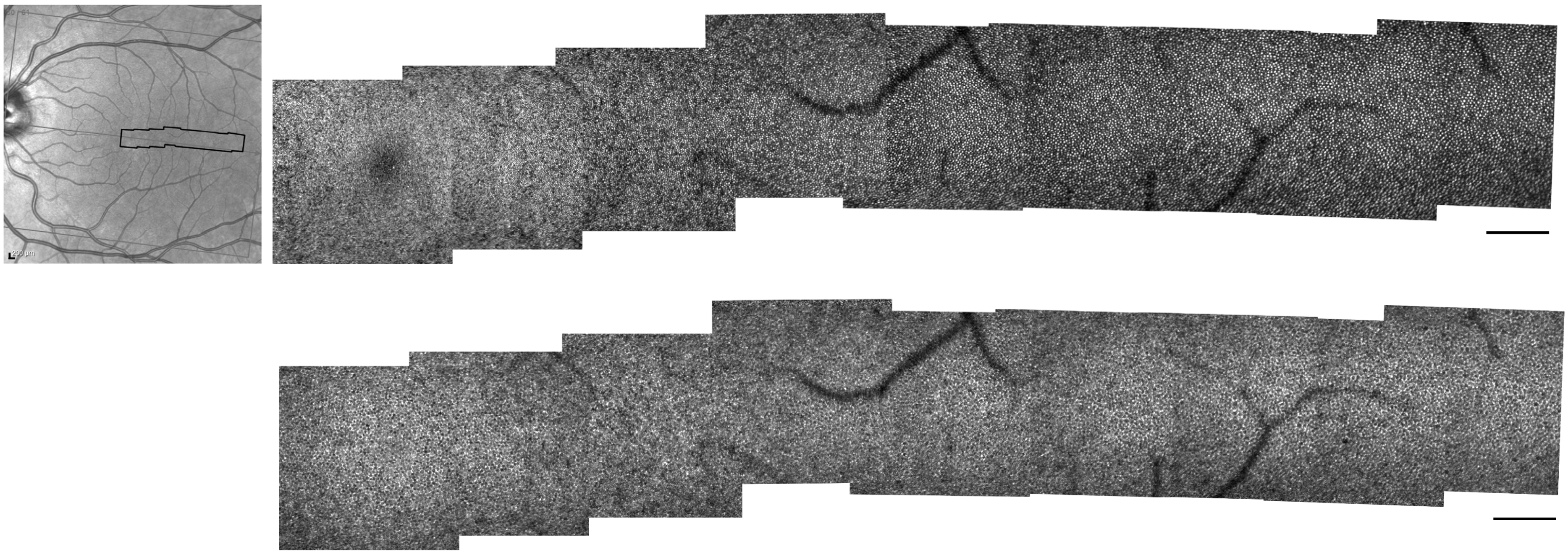

Figure S5. Heidelberg Spectralis SLO image (left) indicating temporal macula region images with AO-OCT (black border) and cone PR (top) and RPE (bottom) mosaics for volunteer 3339.

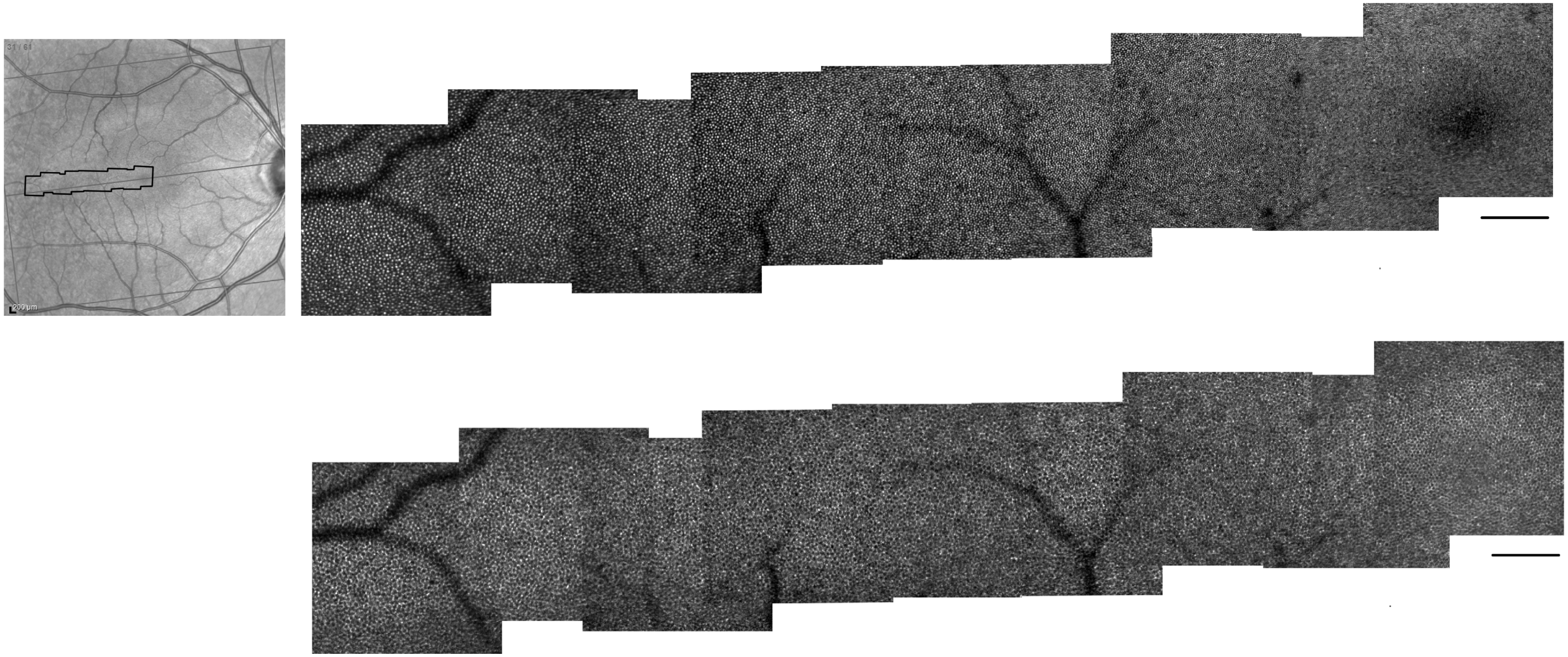

Figure S6. Heidelberg Spectralis SLO image (left) indicating temporal macula region images with AO-OCT (black border) and cone PR (top) and RPE (bottom) mosaics for volunteer 8195.

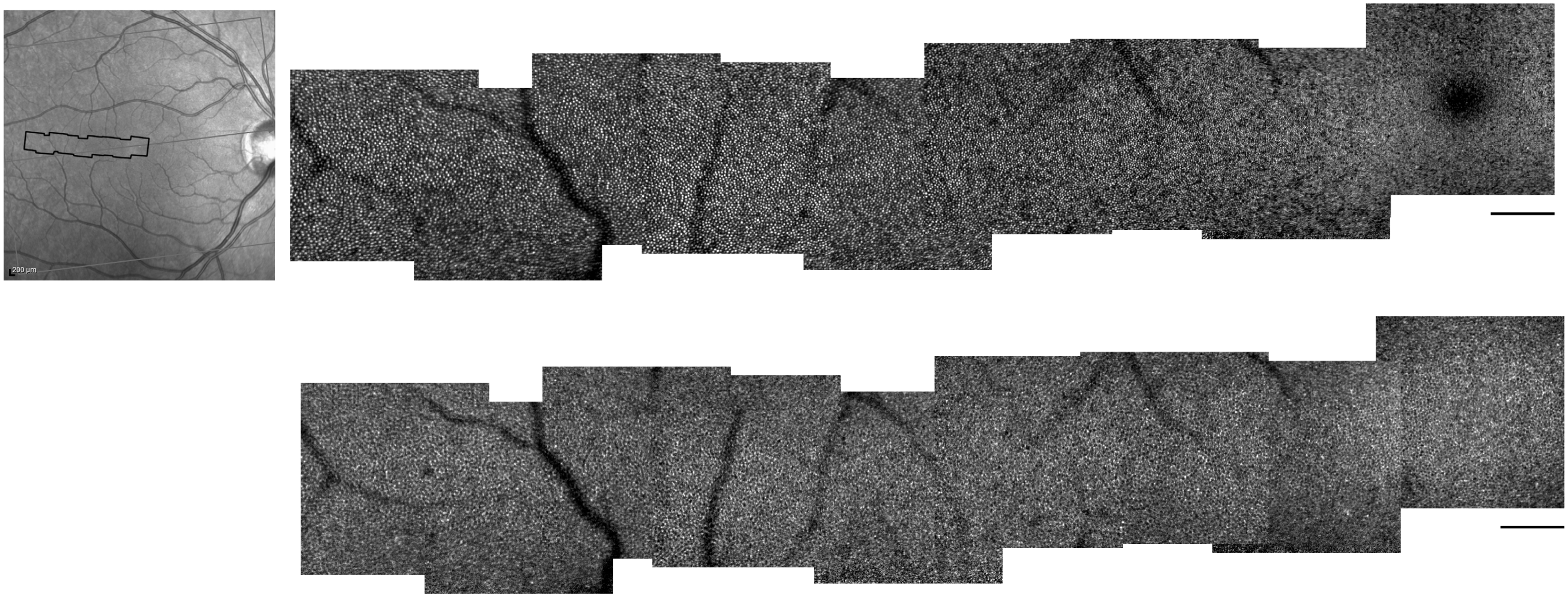

Figure S7. Heidelberg Spectralis SLO image (left) indicating temporal macula region images with AO-OCT (black border) and cone PR (top) and RPE (bottom) mosaics for volunteer 1060.

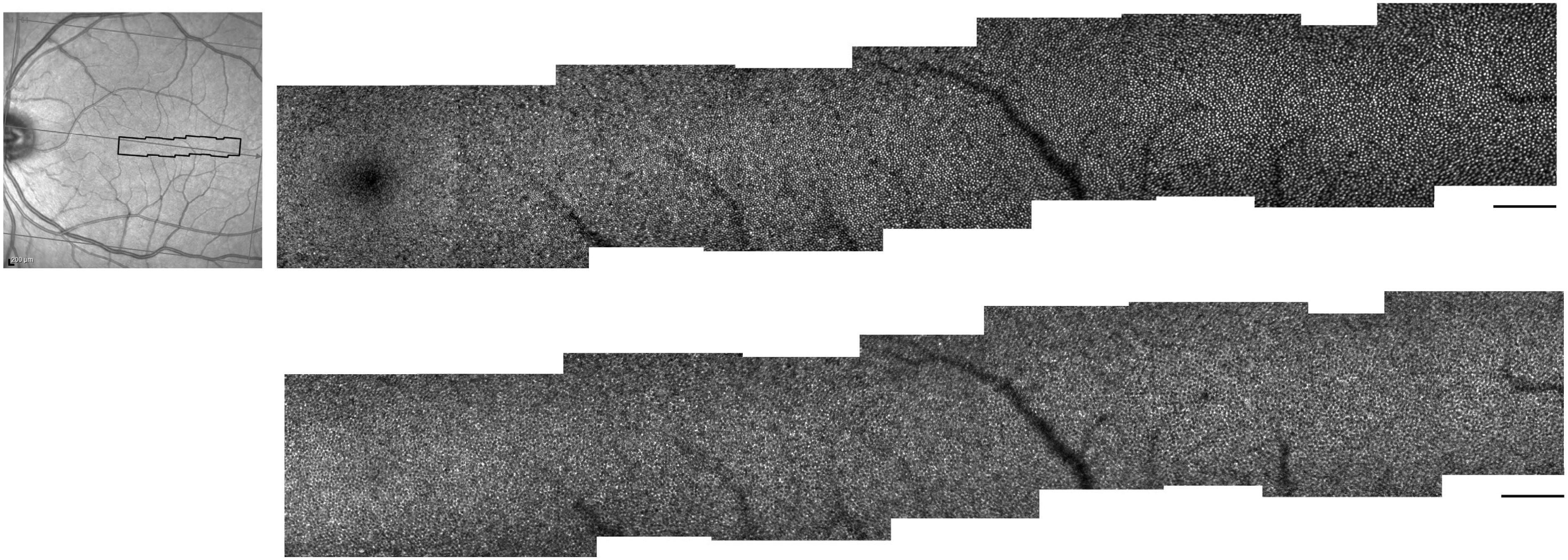

Figure S8. Heidelberg Spectralis SLO image (left) indicating temporal macula region images with AO-OCT (black border) and cone PR (top) and RPE (bottom) mosaics for volunteer 2875.

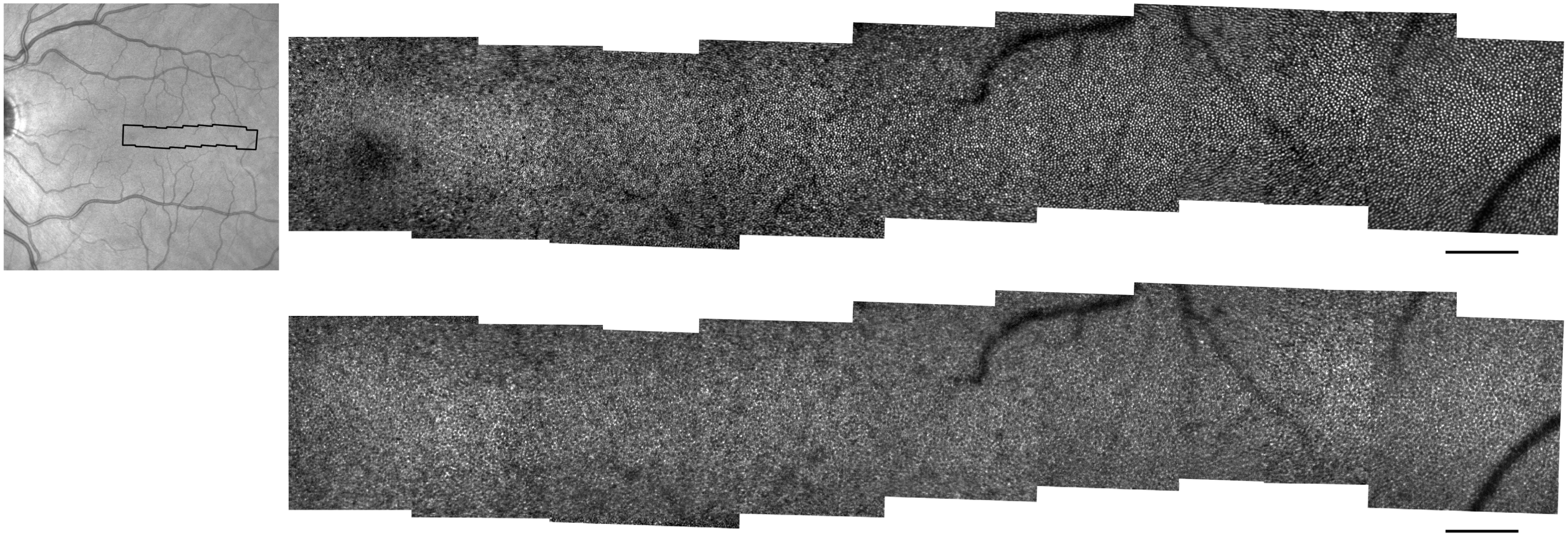

Figure S9. Heidelberg Spectralis SLO image (left) indicating temporal macula region images with AO-OCT (black border) and cone PR (top) and RPE (bottom) mosaics for volunteer 0201.

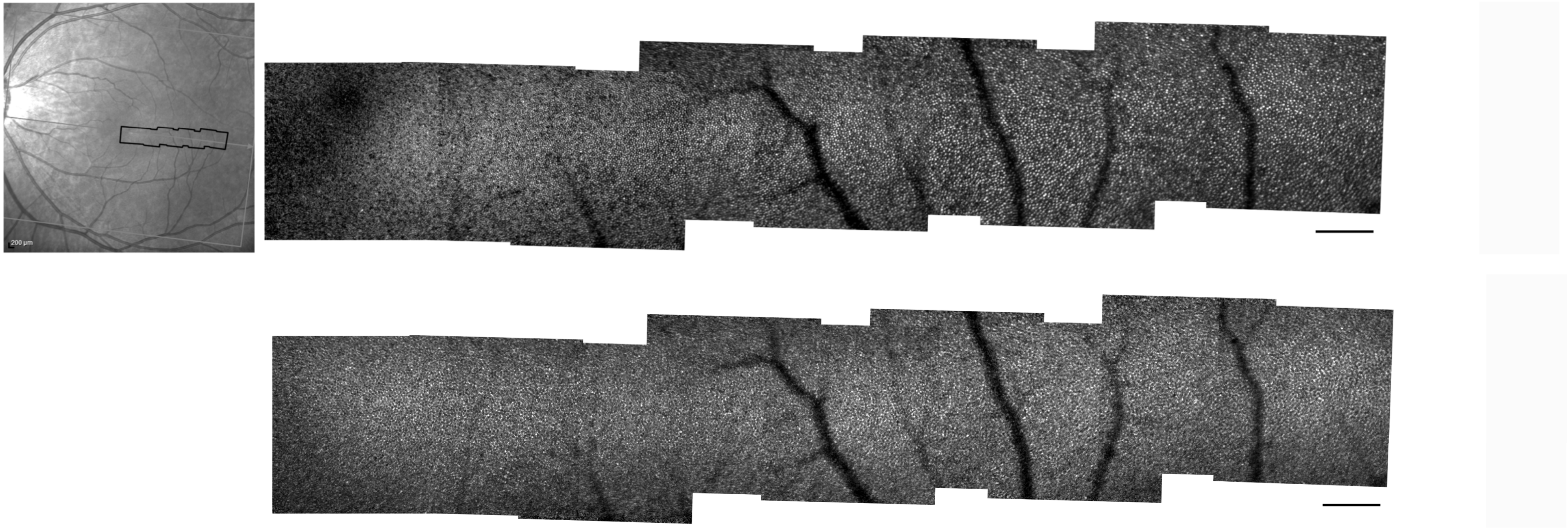

Figure S10. Heidelberg Spectralis SLO image (left) indicating temporal macula region images with AO-OCT (black border) and cone PR (top) and RPE (bottom) mosaics for volunteer 7743.

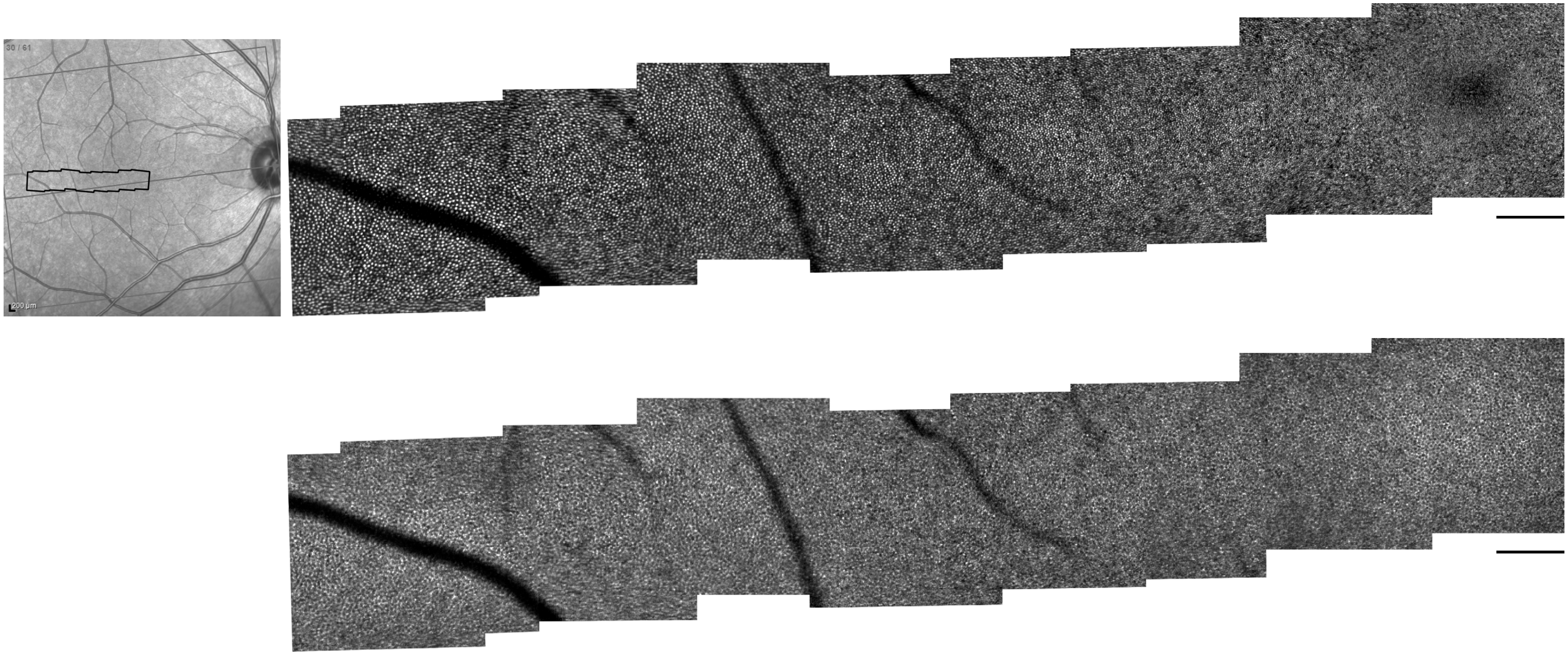

Figure S11. Heidelberg Spectralis SLO image (left) indicating temporal macula region images with AO-OCT (black border) and cone PR (top) and RPE (bottom) mosaics for volunteer 7473.

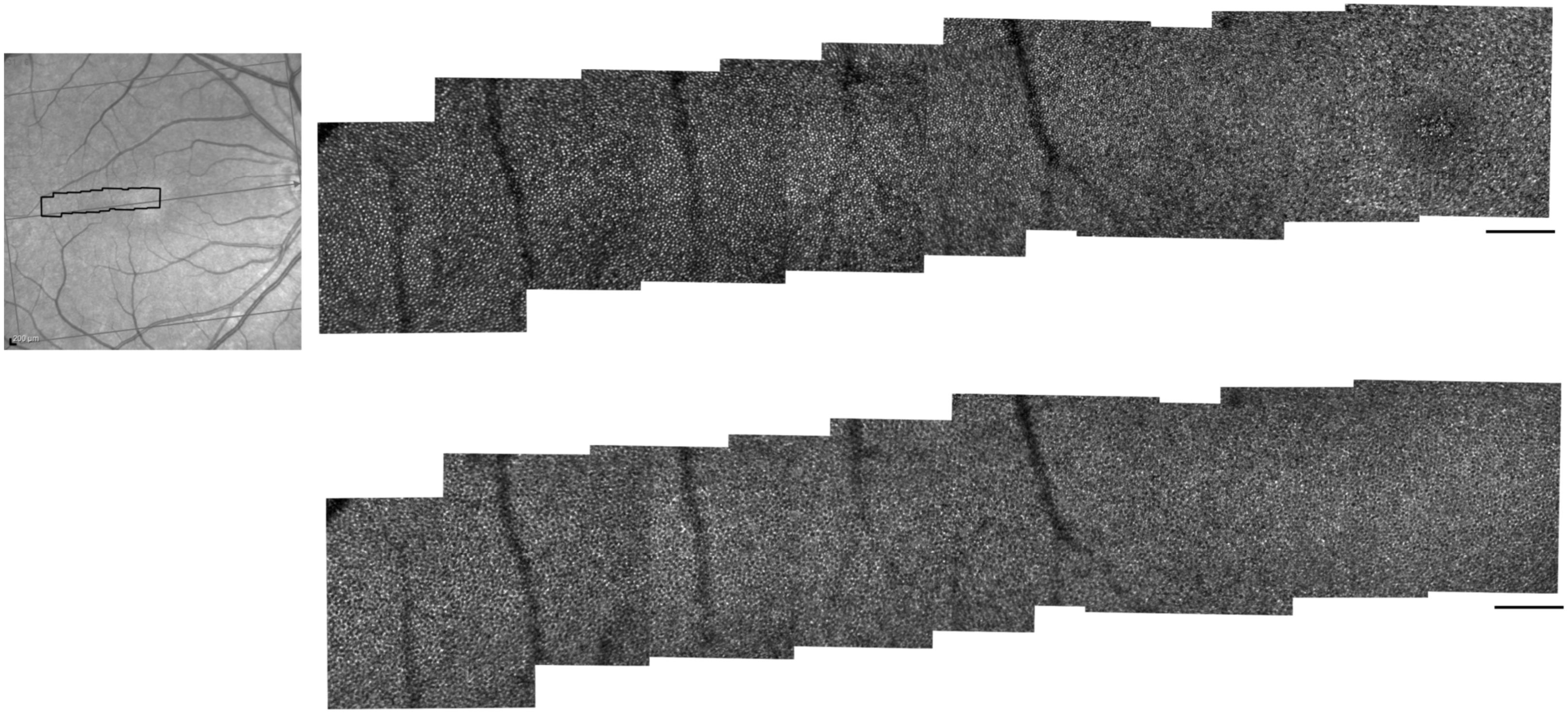

Figure S12. Heidelberg Spectralis SLO image (left) indicating temporal macula region images with AO-OCT (black border) and cone PR (top) and RPE (bottom) mosaics for volunteer 0420.

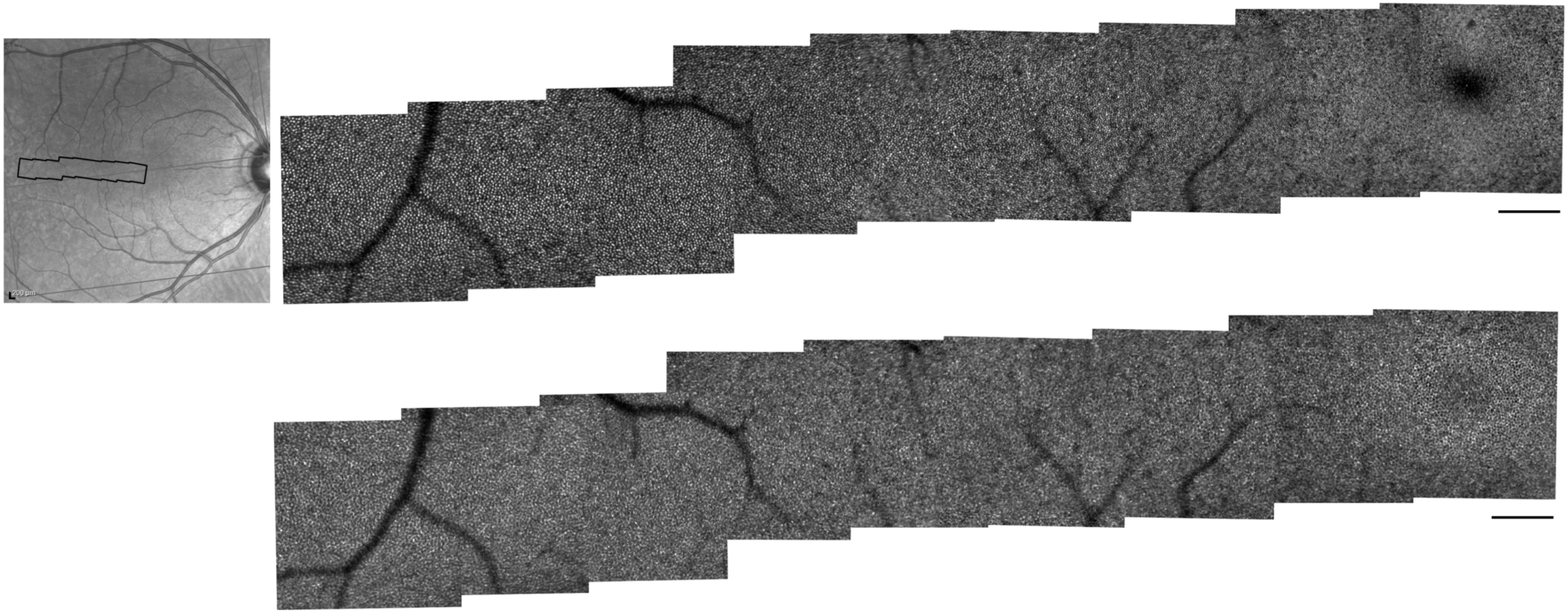

Figure S13. Heidelberg Spectralis SLO image (left) indicating temporal macula region images with AO-OCT (black border) and cone PR (top) and RPE (bottom) mosaics for volunteer 5291.

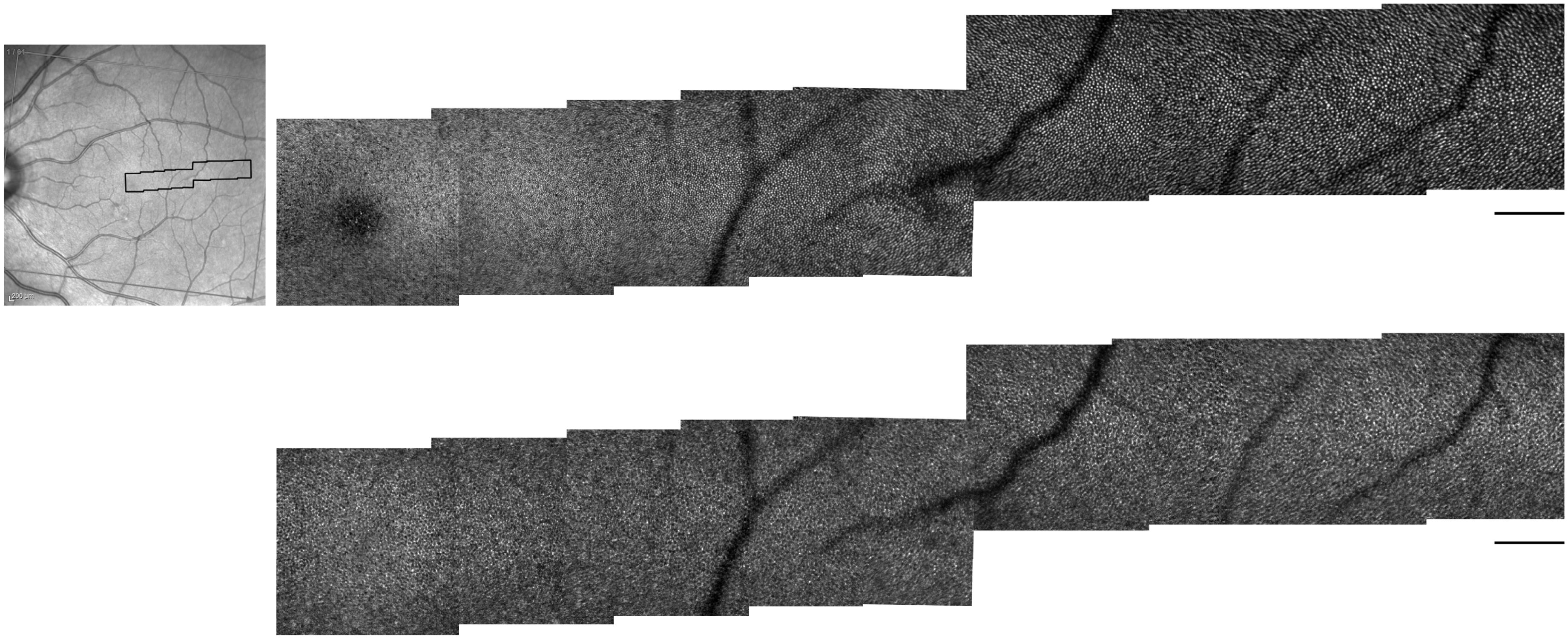

Figure S14. Heidelberg Spectralis SLO image (left) indicating temporal macula region images with AO-OCT (black border) and cone PR (top) and RPE (bottom) mosaics for volunteer 5810.

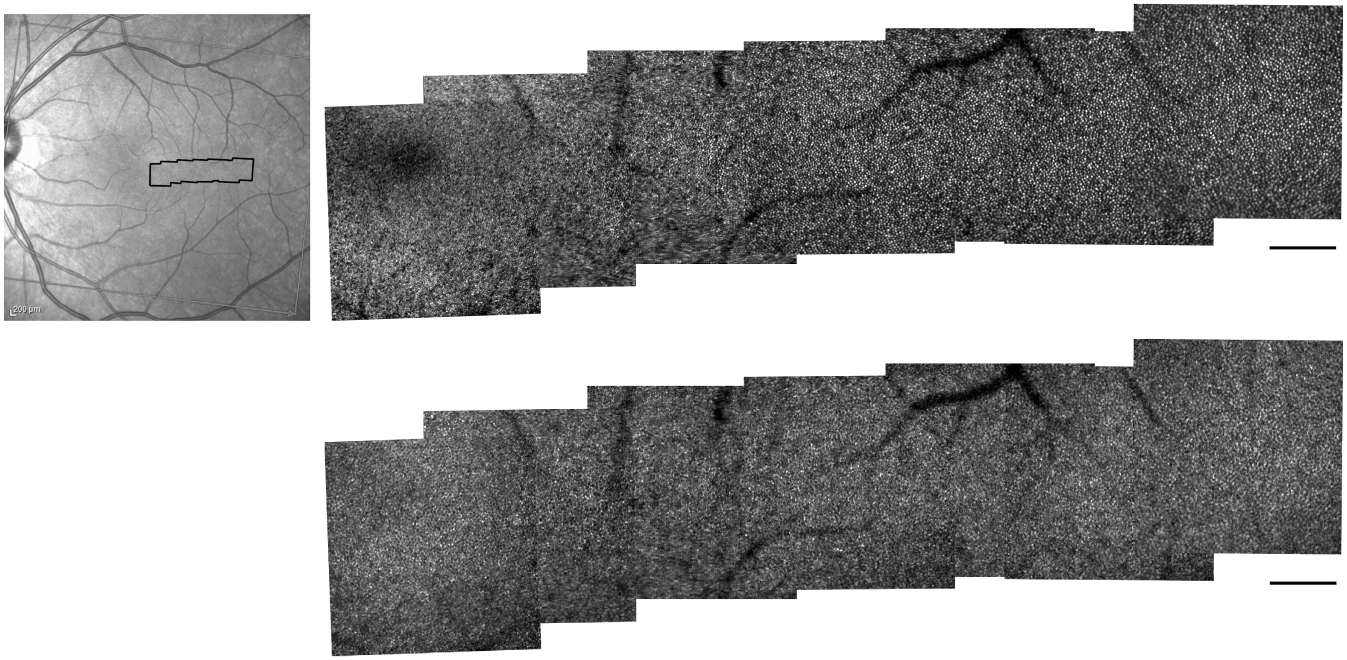

Figure S15. Heidelberg Spectralis SLO image (left) indicating temporal macula region images with AO-OCT (black border) and cone PR (top) and RPE (bottom) mosaics for volunteer 0571.
